# Crystal structures of Mycobacterium tuberculosis GlgE and complexes with non-covalent inhibitors

**DOI:** 10.1038/srep12830

**Published:** 2015-08-06

**Authors:** Jared J. Lindenberger, Sri Kumar Veleti, Brittney N. Wilson, Steven J. Sucheck, Donald R. Ronning

**Affiliations:** 1 Department of Chemistry and Biochemistry, The University of Toledo, 2801 W. Bancroft St. Ms602, Toledo, OH, United States

## Abstract

GlgE is a bacterial maltosyltransferase that catalyzes the elongation of a cytosolic, branched α-glucan. In *Mycobacterium tuberculosis* (*M. tb*), inactivation of GlgE (Mtb GlgE) results in the rapid death of the organism due to a toxic accumulation of the maltosyl donor, maltose-1-phosphate (M1P), suggesting that GlgE is an intriguing target for inhibitor design. In this study, the crystal structures of the Mtb GlgE in a binary complex with maltose and a ternary complex with maltose and a maltosyl-acceptor molecule, maltohexaose, were solved to 3.3 Å and 4.0 Å, respectively. The maltohexaose structure reveals a dominant site for α-glucan binding. To obtain more detailed interactions between first generation, non-covalent inhibitors and GlgE, a variant *Streptomyces coelicolor* GlgEI (Sco GlgEI-V279S) was made to better emulate the Mtb GlgE M1P binding site. The structure of Sco GlgEI-V279S complexed with α-maltose-*C*-phosphonate (MCP), a non-hydrolyzable substrate analogue, was solved to 1.9 Å resolution, and the structure of Sco GlgEI-V279S complexed with 2,5-dideoxy-3-O-α-D-glucopyranosyl-2,5-imino-D-mannitol (DDGIM), an oxocarbenium mimic, was solved to 2.5 Å resolution. These structures detail important interactions that contribute to the inhibitory activity of these compounds, and provide information on future designs that may be exploited to improve upon these first generation GlgE inhibitors.

Tuberculosis (TB) continues to be a global health threat. The World Health Organization estimates that nearly 9.0 million new TB cases were reported, and 1.5 million people succumbed to the disease in 2013^1^. The persistence of TB globally is due in part to the hardiness of the bacterium *Mycobacterium tuberculosis* (*M. tb*), the etiological agent of TB, and the ability of *M. tb* to enter a dormant phase that complicates treatment. Typical therapeutic regimens to treat *M. tb* infections require at least 6 months of anti-tubercular drugs and patient non-compliance during this treatment period is thought to contribute to the selection of antibiotic resistant strains^2^,^3^. In 2013, 1 in 4 cases of TB were multiply drug resistant (MDR-TB), while nearly 1 in 11 of these MDR-TB cases were also extensively drug resistant. Totally drug resistant TB, which is untreatable by current TB drugs, has also emerged^4^. With current therapies becoming ineffective and the high TB burden continuing worldwide, the need for new drug targets and new therapeutics is of paramount importance. One strategy to forestall the selection of resistant strains is to target enzymes whose inhibition leads to rapid killing of both dividing and non-dividing *M. tb*. This would shorten the duration of treatment during the acute infection, thus decreasing the selection of resistant strains. One such example is GlgE, which has been genetically validated as an anti-tubercular target^5^.

The *glgE* pathway of *M. tb* is one of three α-glucan biosynthetic pathways encoded by the *M. tb* genome^6^. This pathway produces a branched, cytosolic glucan using trehalose as a building block through the action of four different enzymes: TreS, Pep2, GlgE, and GlgB (Fig. 1A). GlgE is an α-maltose-1-phosphate:(1 → 4)-α-D-glucan-4-α-D-maltosyltransferase that catalyzes the addition of maltose to maltooligosaccharides (Fig. 1B). GlgE uses M1P to generate the α-1,4-glucan, while GlgB forms α-1,6 branches also using M1P as a substrate.

Deletion of the *glgE* gene in *M. tb* results in the rapid killing of the bacterium due to the toxic effects of M1P accumulation^5^. The increase of M1P concentration elicits an apparent stress response by the bacterium that stimulates the over expression of biosynthetic enzymes necessary for the production of trehalose and more M1P. This positive feedback loop and overproduction of M1P causes pleiotropic effects that cause rapid bacterial death^5^. This effect is novel in that killing is the result of an over production of a toxic metabolite rather than the absence of an important metabolite. Because of this rapid and novel mechanism of killing, efforts to discover GlgE inhibitors may afford the development of potent compounds that rapidly kill *M. tb*.

Several crystal structures of a GlgE ortholog from *Streptomyces coelicolor* (Sco GlgEI) have been elucidated and the enzymatic mechanism characterized^7^,^8^,^33^. It has been shown that Sco GlgEI and Mtb GlgE possess similar kinetic properties and many conserved active site residues. However, enzyme inhibition studies have shown that the Mtb and Sco GlgE orthologs respond differently to inhibition by cyclodextrins, suggesting that the glucan binding site of Mtb GlgE may be different from that of Sco GlgEI. To better understand the molecular basis of the Mtb GlgE enzyme for drug design, and to further characterize the similarities of the Sco and Mtb GlgE orthologs, we have pursued the structure determination of the Mtb GlgE enzyme. Here we report Mtb GlgE structures of a binary complex with maltose and a ternary complex with maltose and maltohexaose, a linear maltooligosaccharide. In addition, a variant of the Sco GlgEI that has an M1P binding site more representative of the Mtb GlgE site was co-crystallized with two different classes of GlgE inhibitors and the X-ray crystal structures were solved.

## Results and Discussion

### Structural comparison of the Mtb GlgE and Sco GlgEI

The crystal structure of the wild type Mtb GlgE bound to maltose (Mtb GlgE-MAL) was solved to 3.3 Å resolution using molecular replacement with the Sco GlgEI structure (RCSB accession number 3ZT5) as the search model (Table 1). Both structures share a highly conserved architecture. Superimposing the homodimers of the Sco GlgEI and Mtb GlgE-MAL using the Cα atoms results in an R.M.S. displacement value of 2.5 Å. Overall, the Mtb GlgE structure is very similar to the previously reported Sco GlgEI enzyme with both enzymes sharing the same 5-domain architecture. Domain A, Insert 1, Insert 2, and Domain B, define the overall catalytic domain and the M1P binding site of the Mtb GlgE. Domain A, Domain N, and Domain S form the very extended dimer interface between GlgE subunits. Finally, Domain C along with Domain S, may play a role in maltosyl-acceptor substrate binding^7^. SAXS studies have demonstrated that both the Sco GlgEI and Mtb GlgE appeared to have similar homodimeric assembly, but the relative orientation of the monomers within a homodimer appears to be slightly different^7^,^8^. In contrast, analysis of the crystal structures described here shows no marked change in the relative orientations of each monomer in the respective homodimer. The differences observed in the homodimer from the SAXS experiments may be attributed to the presence of a disulfide bridge that covalently links the monomers in the Mtb GlgE crystal structure, while this is absent in the Sco GlgEI homolog.

The M1P binding sites of both enzymes are highly conserved in sequence and structure (Fig. 2A). This site is divided into two parts: the −1 subsite where the reducing end of maltose or the phospho-glucosyl moiety of the M1P substrate binds, and the −2 subsite where the non-reducing glucosyl moiety is bound^9^. Both the Mtb and Sco GlgE homologs exhibit a similar set of interactions that coordinate the maltose and position it within the M1P binding site due to the high level of amino acid conservation in the M1P binding site. The only residue that differs between the Mtb GlgE and Sco GlgEI M1P binding sites is represented by S303 and V279, respectively. In both orthologs, the corresponding residue is positioned within the −2 subsite. In the Mtb GlgE, S303 forms a hydrogen bonded interaction with the endocyclic O5 oxygen atom of the −2 glucose in the Mtb GlgE-MAL complex (Fig. 2B). In the Sco GlgEI, the corresponding V279 residue protrudes into the M1P binding site in the same orientation as the Mtb GlgE S303 residue (Fig. 2B). Rather than forming a hydrogen bond with substrate, V279 of Sco GlgEI forms a van der Waals interaction with two carbon atoms in the ring bound in the −2 subsite. This difference affects neither the active site structure nor the *K*_M_^app^ for M1P observed for Mtb GlgE, Sco GlgEI, or Sco GlgEI-V279S^5^,^7^,^10^.

As previously stated, one additional minor difference between the Sco and Mtb GlgE orthologs is that the Mtb GlgE crystal structures possess an intermolecular disulfide bridge between residue C29 of the two molecules in the GlgE dimer. The C29 residue is not conserved in the majority of the deposited GlgE protein sequences and GlgE orthologs form a stable homodimer when lacking a disulfide bond. Therefore, it is unlikely that the observed disulfide bond plays any important physiological role.

### A dominant binding site for a linear glucan revealed by Mtb GlgE complexed with maltohexaose

GlgE utilizes an α-1,6-branched, α-1,4 glucan as a maltosyl-acceptor molecule and thereby extends the linear portion of the glucan using M1P as the maltosyl donor. To further characterize the maltosyl acceptor binding site and the mode of GlgE binding to linear polysaccharides, we soaked Mtb GlgE-MAL co-crystals with maltohexaose immediately prior to performing X-ray diffraction experiments. A crystal structure of a ternary complex with Mtb GlgE bound to maltose and maltohexaose (GlgE-M6) was solved to 4.0 Å resolution (Table 1). Initial inspection of the Fo-Fc maps calculated following rigid body refinement revealed a horseshoe-shaped density that was more than six times above background^11^,^12^. This density, when fit with the maltohexaose ligand, corresponded to a single molecule of maltohexaose (Fig. 3A).

The maltohexaose binds to multiple loop regions of Domains A and C that define a portion of the acceptor site; specifically, maltohexaose binds the short loop regions that connect secondary structural elements encompassing residues 465–481, 504–514, and 622–632 (Fig. 3B). Specific binding interactions between the Mtb GlgE and maltohexaose are mediated primarily through hydrogen bonding interactions with the backbone atoms of residues that encompass the acceptor site, including the backbone carbonyls of T474, N512, and L628. Two amino acid side chains also contribute to maltohexaose binding. Specifically, residue N629 in Domain A forms a hydrogen bond through the carbonyl of the carboxamide moiety, while the side chain of F631 forms van der Waals interactions with the fourth sugar of the maltohexaose^13^. The dearth of specific side chain interactions with maltohexaose suggests that conservation of the structure of the protein backbone atoms at this site is required for glucan binding, but amino acid sequence conservation may not be as important (Fig. 3B,C). Comparing the residues of the maltohexaose binding sites in the Sco GlgEI and Mtb GlgE structures exhibits approximately 74% sequence identity between the two proteins. The amino acid differences between the two enzymes within this site include residues T474, N512, and F631 of the Mtb GlgE, which are represented by residues N450, G489, and R606 in the Sco GlgEI enzyme, respectively. Based on the interactions observed in the Mtb GlgE-M6 structure, the variation in side chain identity for these three residues should not significantly impact maltohexaose binding since the interactions are mediated by backbone atoms (T474 and N512), or through non-specific van der Waals interactions (F631).

Surprisingly, the maltohexaose is situated approximately 26 Å from the M1P binding site; a distance that requires approximately three additional maltohexaose units to span. Therefore, the high affinity maltohexaose binding site observed in the Mtb GlgE-M6 structure likely represents only a small portion of a much larger α-1,6-branched, α-1,4 glucan binding site. While sequence conservation between the Mtb and Sco GlgE enzymes at the maltohexaose site is only 74%, the surface leading from the high affinity site into the enzyme active site are nearly identical in both amino acid sequence and three dimensional structural. This suggests that this surface region is very important for correctly orienting the large glucans representing the maltosyl acceptor substrate.

It had been shown previously that cyclodextrins inhibit the Sco GlgEI weakly, but show no inhibition towards the Mtb GlgE. Structures of the Sco GlgEI bound to both α-cyclodextrin (IC_50_ = 19 mM) and β-cyclodextrin (IC_50_ = 6 mM) have been solved (RCSB accession numbers: 3ZT6 and 3ZT7)^7^. In these structures, the cyclodextrins sit at the homodimer interface in a largely hydrophobic site formed by Domain A of one subunit and Domain N of the second subunit. The site is orthogonal to the M1P binding site and adjacent to the observed maltohexaose binding site as seen in the Mtb GlgE-M6 structure, but the maltohexaose and cyclodextrin binding sites do not overlap (Fig. 3C). It was hypothesized that cyclodextrin inhibition of the Sco GlgEI is likely due to partial occlusion of the acceptor glucan binding site and prevention of the glucan substrate from accessing the enzyme active site^7^.

The Mtb GlgE structures presented here offer further insight to the differences between the Sco and Mtb GlgE orthologs that likely preclude binding of the cyclodextrins to the Mtb GlgE. The observed cyclodextrin binding site in Sco GlgEI differs from the Mtb GlgE ortholog at two amino acid positions: residues R427 in one protein subunit and G84 from the neighboring subunit of the Sco GlgEI are both replaced by proline residues in the Mtb GlgE structure. The resulting structural changes within this region likely abolish cyclodextrin binding to Mtb GlgE due to a potential steric clash between the proline side chains and the cyclodextrin. Although Mtb GlgE is not inhibited by cyclodextrins and is unlikely capable of high affinity cyclodextrin binding at the same site observed in the Sco GlgEI ortholog, the cyclodextrin binding site observed in the Sco GlgE does indeed appear to represent a small portion of the conserved path leading from the maltohexaose binding site observed in the Mtb GlgE-M6 structure to the M1P binding site (Fig. 3C).

### The Sco GlgE-V279S variant as a model to mimic the Mtb GlgE active site

The conserved domain structure of the Mtb GlgE and Sco GlgEI enzymes and the similar kinetics of the maltosyltransfer reaction catalyzed by both enzymes, suggest that Sco GlgEI is a reasonable surrogate for developing and testing potential Mtb GlgE inhibitors^7^. Since crystals of the Sco GlgEI are known to diffract to a much higher resolution than crystals obtained with the Mtb GlgE, which is essential to elucidate the detailed interactions between proteins and bound inhibitors, an Sco GlgEI variant was constructed that would afford high-resolution X-ray diffraction data and possess an M1P binding site identical to that of the Mtb GlgE. This is particularly important for future GlgE inhibitor development, since the hydrophobicity of a region targeted for inhibition is an important characteristic that defines the druggability of an enzyme^14^,^15^. Specifically, the presence of V279 in the Sco GlgEI M1P binding site significantly increases the hydrophobicity of this site when compared to the Mtb GlgE enzyme, which possesses a serine residue at the analogous site (Fig. 2B). Therefore, a mutation was made to the *S. co glgEI* gene to encode a V279S variant of Sco GlgEI. This variant perfectly matches the M1P binding site observed in the Mtb GlgE structure and has already been shown to retain its maltosyltransferase activity and is inhibited by compounds shown to affect Mtb GlgE enzymatic activity^10^.

### Crystal structure of Sco GlgEI-V279S in complex with a non-hydrolyzable M1P analogue

**Figure 1:**
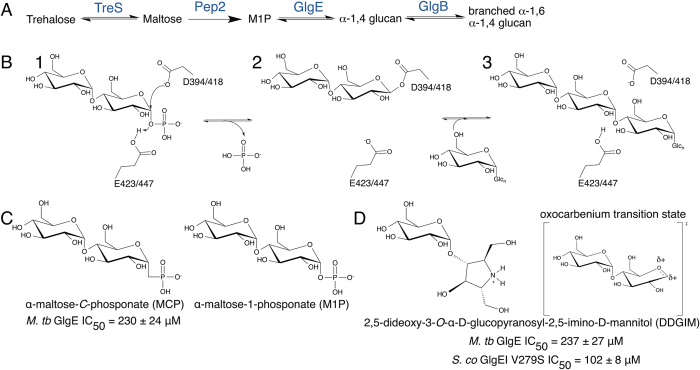
α-1,4 glucan biosynthetic pathway, catalytic mechanism of GlgE, and current inhibitors of GlgE activity. (**A**) Biosynthetic pathway of the cytosolic α-1,4 glucan: trehalose is isomerized to maltose (TreS), which is subsequently phosphorylated (Pep2) to produce maltose-1-phosphate (M1P). M1P is used as the maltosyl donor in the generation of the liner glucan (GlgE) or branched α-1,6 glucan (GlgB). (**B**) GlgE mechanism. (1) Protonation by the general acid leads to the loss of phosphate and formation of the maltosyl enzyme intermediate. (3) Deprotonation of the 4-OH of the acceptor leads to the transfer of the maltose unit to the acceptor. (**C**) Structure and inhibitory data of a non-hydrolysable substrate analogue inhibitor of GlgE, α-maltose-*C-*phosphonate (MCP) next to the natural substrate α-maltose-1*-*phosphate (M1P) and (**D**) a transition-state mimic of GlgE, 2,5-dideoxy-3-*O*-α-D-glucopyranosyl-2,5-imino-D-mannitol (DDGIM) next to the oxocarbenium transition-state.

**Figure 2:**
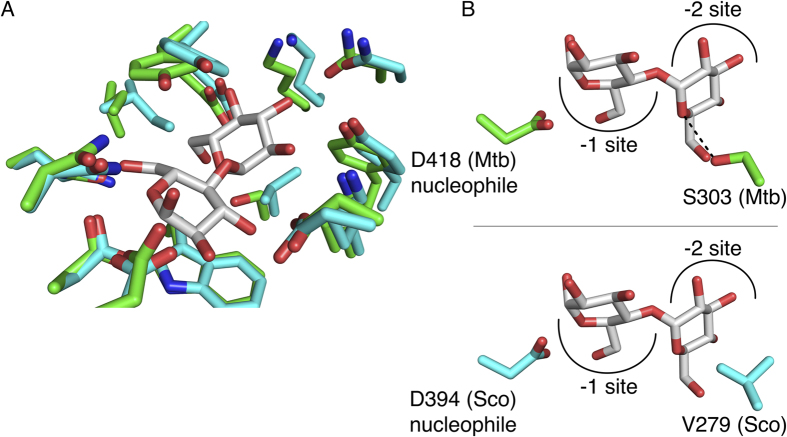
Mtb GlgE M1P donor site. (**A**) The M1P donor sites of the Mtb GlgE (green) and Sco GlgEI (cyan) with the active site nucleophile and maltose bound. (**B**) Both donor sites are highly conserved with the only difference being the presence of a serine (S303) in the Mtb GlgE, while a valine (V279) is present in the Sco GlgEI.

Since GlgE uses M1P as a maltosyl donor to extend the glucan, it was hypothesized that a non-hydrolyzable substrate analog could potentially function as a competitive inhibitor of GlgE. This compound, α-maltose-*C*-phosphonate (MCP), was synthesized and tested for GlgE inhibitory activity (Fig. 1C)^16^. MCP inhibited the Mtb GlgE with an IC_50_ of 237 ± 24 μM, which is roughly equivalent to the *K*_M_ of the natural M1P substrate. This compound was co-crystallized with the Sco GlgEI-V279S to characterize the molecular basis of this inhibition. The resulting 1.9 Å resolution structure (Table 1) exhibits difference density within the M1P binding site that clearly illustrates the binding mode of MCP (Fig. 4A).

Throughout the remainder of this manuscript, residue numbering is formatted where the number preceding the backward slash represents that of Sco GlgEI and the number following the backward slash represents the residue number of the Mtb GlgE. Coordination of the phosphonate moiety is achieved by four residues in the phosphate binding site of the maltosyl donor pocket: N395/419, K355/379, N352/376, and Y357/381 (Fig. 4B). The side chain of K355/379 forms an ionic interaction with the negatively charged OP2 of the phosphonate moiety, while the Nδ atom of the N352/376 side chain and the hydroxyl of Y357/381 coordinate the carbonyl of the phosphonate (OP1) via hydrogen bonding interactions. Lastly, the carbonyl oxygen of the N395/419 side chain forms a hydrogen bond with the third oxygen atom of the phosphonate moiety (OP3), suggesting that this oxygen is protonated (Fig. 4B).

Since it was not necessary to make an active site mutant to form the stable GlgEI-V279S-MCP complex, this structure exhibits the direct interactions between the nucleophilic D394/418 residue and the M1P substrate immediately prior to nucleophilic attack. In this structure, D394/418 is positioned 3.3 Å from the C1′ atom of MCP. Other important interactions of the −1 subsite seen in the GlgEI-V279S-MCP complex have been described in previously published GlgEI structures^7^,^8^. Briefly, the Nδ atom of N395/419 is hydrogen bonded to the endocyclic oxygen of the −1 sugar. An additional hydrogen bond is formed between Q324/348 and the O6′ hydroxyl group, as well as between D480/504 and O3′ of the MCP. The Oε atom of E423/447 was proposed to function as a general acid to protonate the phosphate leaving group during the first step of the reaction and is positioned approximately 3.8 Å from the carbon of the phosphonate moiety that has replaced the bridging oxygen of the M1P substrate. Other interactions seen here are R392/416 and the O2′ hydroxyl as well as hydrogen bonds between the O6′ and D394/418 and Q324/348. Additionally, the glucosyl moiety in the −2 subsite forms an extensive hydrogen bonded network with the side chains of residues K264/288, N268/292, Y535/559, D359/383, S279/303, and K534/558 as well as the backbone amide of A282/306 (Fig. 4B) as seen and described in previous Sco GlgEI structures^7^,^8^.

The MCP bound complex structure gives the first complete picture of the fully intact and functional GlgE active site in the presence of a substrate analog. The MCP bound structure described here shows many of the same interactions as those described in the Sco GlgEI-D394A-M1P (RCSB accession number: 4CN1), but one interesting change was observed. The ionic interaction between the side chain ammonium of K355 and the OP1 of the MCP structure has a bond distance of 2.9 Å, while the equivalent bond in the M1P bound structure is much weaker at a distance of 4.3 Å. This could simply be a function of the slightly improved density for the phosphonate moiety as compared to the phosphate of the Sco GlgEI-D394A-M1P structure. Alternatively, the increase in distance may be the result of slight structural alterations of the active site in the inactive Sco GlgEI-D394A as a result of the mutation of the nucleophile, D394, to an alanine residue. Regardless, the intact active site observed in the MCP structure described here may be slightly more representative of interactions that occur between the enzyme and substrate.

Comparing the structures of the Mtb GlgE-MAL complex and the Sco GlgEI-V279S-MCP complex shows little change within the enzyme active sites. Despite the absence of the phosphate in the phosphate binding site of the GlgE-MAL, the residues responsible for this coordination exhibit only minor positional shifts when compared to the maltose-bound structure. This suggests a structural rigidity in the active site that will improve the probably of designing improved inhibitors.

It has previously been shown that the GlgE enzymes are unable to use α-glucose-1-phosphate as a donor molecule^7^. Because the sole difference between the phospho-sugars is the presence of the additional glucose of the M1P, it would appear that the −2 subsite is extremely important for substrate binding and recognition, and subsequent catalysis. Indeed, the −2 subsite contributes many more hydrogen bonding interactions than the −1 subsite. Additionally, the mutation of the Sco GlgEI to the V279S variant adds an additional hydrogen bond between the serine side chain and the endocyclic oxygen of the second glucosyl moiety in the −2 subsite. Therefore, the design of the next generation of inhibitors should continue to exploit the hydrogen bonded interactions observed in the −2 subsite.

### Crystal structure of Sco GlgEI-V279S in complex with an oxocarbenium mimic

GlgE belongs to the GH13_3 subfamily of glycoside hydrolase enzymes that proceeds through an oxocarbenium^5^,^7^. As such, both intermediate and transition state analogues have been used as potent inhibitors of these hydrolytic enzymes^17^,^18^,^19^. Therefore, we synthesized and tested an azasugar, 2,5-dideoxy-3-*O*-α-D-glucopyranosyl-2,5-imino-D-mannitol (DDGIM), that would mimic the oxocarbenium formed through a step-wise dissociative mechanism (Fig. 1D)^20^. The DDGIM mimics both the positive charge at the corresponding position, as well as the half-chair conformation^10^,^21^,^22^. The DDGIM showed inhibition of both the Mtb GlgE and the Sco GlgE-V279S with IC_50_ values of 237 ± 27 μM and 102 ± 8 μM, respectively^10^,^16^. To begin characterizing the inhibitory mechanism, DDGIM was co-crystallized with Sco GlgEI-V279S and the resulting crystal structure refined to 2.5 Å resolution (Table 1).

Inspection of the initial Fo-Fc map following rigid body refinement clearly showed the presence of the compound in the enzyme active site. Refinement of the GlgEI-V279S-DDGIM co-crystal structure shows the five membered imino mannitol moiety occupying the −1 subsite of the active site with numerous interactions orienting the imino mannitol (Fig. 4C). The O1′ hydroxyl group is forming hydrogen bonded interactions with the side chains of both R392/416 and E423/447, the general acid in the first step of the catalytic cycle. The nucleophile D394/418, forms an ionic interaction with the secondary ammonium of the imino mannitol moiety at a distance of 2.6 Å, which is significantly shorter than the observed bond distance between the D394/418 and the C1′ of the MCP bound structure (3.3 Å). The glucose moiety of DDGIM bound in the −2 subsite forms an extensive hydrogen bonded interaction network the same as that seen in previous GlgE structures (Fig. 4D).

It is intriguing that, although the five-member imino mannitol ring contains one less hydroxyl group and lacks a phospho-mimicking moiety, DDGIM exhibits an IC_50_ value (Mtb GlgE = 237 ± 27 μM and Sco GlgE = 102 ± 8 μM) similar to that observed for the MCP substrate analog (Mtb GlgE 250 ± 24 μM). Chemical properties of DDGIM most likely contributing to the comparable inhibition are the shape and the ionic interaction between the imino mannitol and the D394/418 side chain. The DDGIM mimics the semi-planar, half-chair conformation adopted by the oxocarbenium in the −1 subsite during catalysis. Because of this structural mimicry, the secondary ammonium of the imino mannitol is positioned much closer to the D394/418 nucleophile than the analogous C1′ atom in the MCP bound structure. This newly formed ionic interaction observed between the positively charged secondary ammonium and the nucleophile represents a new interaction not previously observed in any of the binary GlgE enzyme ligand-complexes. Similar to what was observed in previous structures and the MCP complex structure, the numerous interactions between the residues composing the −2 subsite and the glucosyl moiety remain fixed despite the different interactions observed between DDGIM and the −1 subsite. Similar to the conclusions from the MCP bound structure, it appears that maintaining the −2 subsite interactions are vital for binding of substrate and inhibitors, while the enzyme is able to accommodate structural variation of inhibitors within the −1 subsite.

## Conclusions

**Figure 3:**
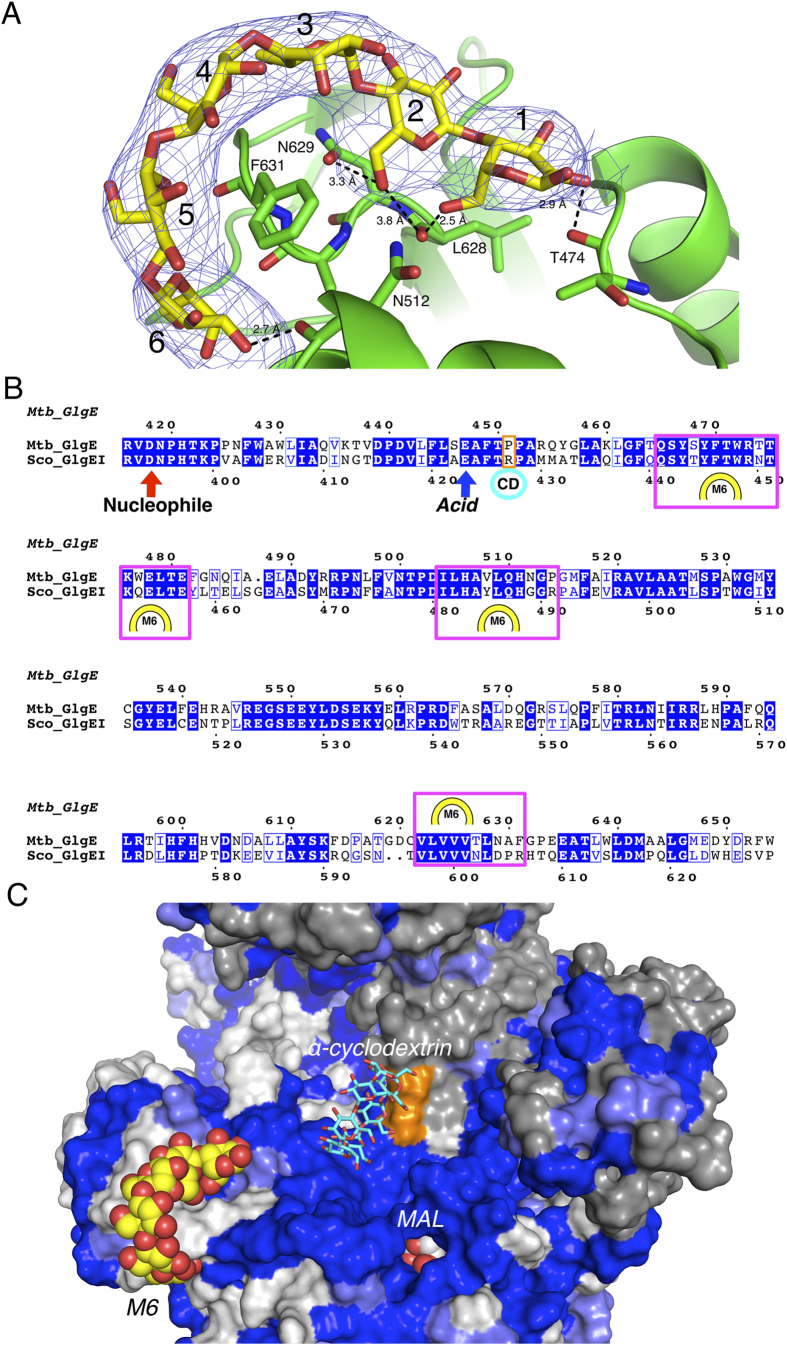
The high-affinity maltohexaose binding site of the Mtb GlgE. (**A**) Fo-Fc omit map calculated while omitting maltohexaose is contoured at 3σ showing the binding of maltohexaose (M6) to the Mtb GlgE. Binding is mediated by backbone carbonyls of T474 of the B domain, N512 and L628 of the A domain, and the carbonyl of the sidechain carboxamide group of N629 of the A domain. F631 of the A domain interacts with the fourth sugar via van der Waals interactions. (**B**) Sequence alignment between Sco GlgEI and Mtb GlgE. Areas defined by the magenta boxes and the M6 symbol indicate the residues forming the maltohexaose binding site observed in Mtb GlgE, while the teal circle indicates the cyclodextrin binding surface observed in Sco GlgEI. The orange box indicates residues important for binding cyclodextrin in Sco GlgEI but differ in Mtb GlgE. (**C**) Surface conservation comparison of the Mtb and Sco GlgE enzymes. Blue surface indicates identical residues, light-blue semi-conserved changes, and white no sequence similarities. The second subunit of the GlgE dimer uses grey to indicate lack of sequence similarity. The orange surface represents changes observed at the cyclodextrin binding pocket. Maltohexaose is shown as yellow spheres and α-cyclodextrin is shown with cyan bonds. Sequence alignment was performed using ESPript 3.0 (http://espript.ibcp.fr)^32^.

**Figure 4:**
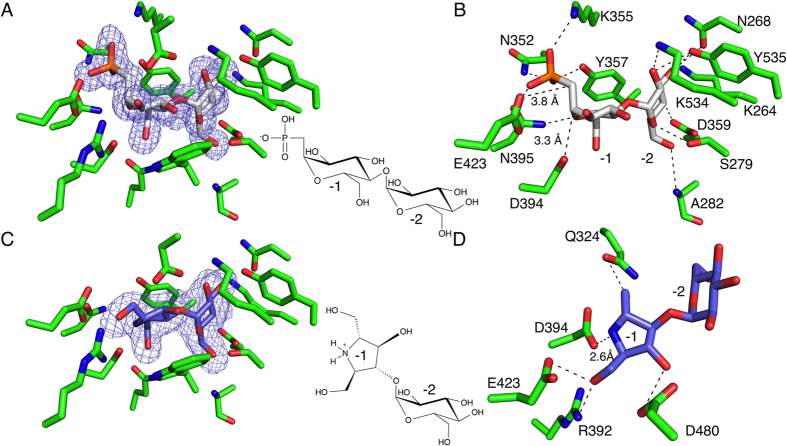
Sco GlgEI-V279S in complex with MCP and DDGIM. (**A**) Fo-Fc omit map calculated while omitting MCP is contoured at 3σ with MCP bound in the enzyme active site. (**B**) Numerous hydrogen bonded interactions coordinate the glucosyl moiety in the −2 site, while the four residues in the phosphate-binding site coordinate the phosphonate moiety of the MCP. The D394/418 nucleophile is present in this structure and is positioned 3.3 Å from the C1′ of the MCP. E423/447 is well positioned to act as a general acid to protonate O1 of the phosphate of M1P at a distance of 3.8 Å. (**C**) Fo-Fc omit map calculated while omitting DDGIM is contoured at 3σ showing the DDGIM bound in the enzyme active site. (**D**) The glucose moiety of the DDGIM in the −2 site is coordinated in the same manner as was observed in the previous structures. Interactions of the imino mannitol in the −1 site are numerous. The O01 hydroxyl is hydrogen bonded with Q324/448, the general acid E423/447 is forming a hydrogen bond via the O01 hydroxyl of the imino mannitol, as well as an additional hydrogen bond between D480/504 and O09 hydroxyl. D394/418 is forming an ionic interaction with the secondary ammonium of the DDGIM. Inset between each image is the line structure of each inhibitor.

Despite the sequence and kinetic similarities between the Sco GlgEI and the Mtb GlgE, elucidating the structure of the Mtb GlgE afforded the full assessment of the similarities and differences between these orthologs. As expected, the Mtb GlgE and Sco GlgEI structures are very similar overall with the Mtb GlgE possessing the same 5-domain architecture observed in the Sco GlgEI. However, the identification of a maltooligosaccharide binding site in Mtb GlgE distinct from the previously identified cyclodextrin binding site for Sco GlgEI was unexpected and highlights some of the inherent challenges in identifying or targeting the binding sites for large biopolymers such as glucans, nucleic acids, and proteins^23^,^24^. The Mtb GlgE and Sco GlgEI orthologs share some sequence identity at the maltohexaose binding site (Figs 3B,C). However, binding at this site appears to be mediated primarily by backbone interactions, which minimizes the importance of sequence conservation as long as backbone structure is conserved (Fig. 3A). It is also likely that the semi-conserved, high affinity maltohexaose binding site represents only a portion of the much larger glucan binding surface of GlgE indicated by the conserved surface region leading from the maltohexaose binding site to the active site (Fig. 3C). Although kinetic experiments indicate that affinity for maltooligosaccharides appears low, it is possible that weak interactions between the conserved surface and the glucan substrate support appropriate positioning to afford nucleophilic attack on the maltosyl-enzyme intermediate formed during catalysis.

However, due to the limitations of the low resolution achieved with the Mtb GlgE structures, care must be taken in the interpretations of structural details. Using the high-resolution Sco GlgE structures as references, appropriate geometry could be obtained for the overall Mtb GlgE structures. When looking at ligand-protein interactions, it becomes slightly more difficult in interpreting interactions, as many of the atoms are poorly resolved in the structure. In the case of the Mtb GlgE-M6 crystal structure, the location of the maltohexaose binding is known with high confidence due to the strong density corresponding very well to the full length malto oligosaccharide. However, given the modest resolution of the maltohexaose structure, the orientation of the maltohexaose cannot be unambiguously defined until a higher resolution structure is determined. Analogously, the Mtb GlgE atoms interacting with maltohexaose are correct but the specific interactions with maltohexaose atoms described here would differ if the orientation of maltohexaose is flipped with respect to the current model.

One important difference between Sco GlgEI and Mtb GlgE that potentially affects inhibitor development is the single amino acid change in the M1P binding site. While Sco GlgEI possesses a valine at position 279, the Mtb GlgE has a serine at the corresponding position (S303). Because the hydrophobicity of the active site is important for inhibitor design, the mutation to produce the V279S variant of the Sco GlgEI is important for future drug discovery efforts. This model Sco GlgEI-V279S variant provided high quality MCP and DDGIM structures that exhibit interactions analogous to that expected to be observed in the Mtb GlgE active site.

Both the MCP and DDGIM structures represent initial design efforts of non-covalent GlgE inhibitors and highlight regions of the enzyme active site where inhibitor design changes should improve inhibitory activity. The MCP represents a non-hydrolyzable substrate analogue and exhibits modest inhibition as expected for a substrate analog (IC_50_ = 237 ± 24 μM). We were able to accurately resolve the interactions between the substrate analog and the active site residues with the catalytic nucleophile present. The DDGIM represents the first attempt at inhibiting GlgE using an oxocarbenium mimic (IC_50_ = 237 ± 27 μM). The shape of the DDGIM and the observed ionic interaction between the nucleophile and secondary ammonium represents a newly observed interaction that can be used for the design of future inhibitors. Despite the structural and chemical variation of the different functional groups that occupy the −1 subsite in the two different inhibitor co-crystal structures, both inhibitors exhibit roughly the same IC_50_ values. This may be due to conserved interactions observed in the −2 subsite of the enzyme active site and suggests that GlgE can likely tolerate a variety of functionalities in the −1 subsite as long as the interactions of the −2 subsite are maintained. This is consistent with chemical and structural changes observed in the maltosyl donor during catalysis and affords the use of a variety of pharmacophores that could bind within the −1 subsite. Additionally, given the structural and chemical similarity of the inhibitors to the M1P substrate in terms of the number of hydrogen bonding and ionic interactions, it is logical that the IC_50_ values are similar to the known *K*_m_ of the M1P substrate being mimicked by these inhibitors. Efforts leveraging this information should provide ideas for the future design of next generation of GlgE inhibitors with significantly improved affinity for the Mtb protein, improved inhibitory activity, and more drug-like properties.

## Methods

### Molecular Cloning

*M. tb* genomic DNA was used to PCR amplify *glgE* (Rv1327c) using the following primers: 5′- CAC CAT ATG AGT GGC CGG GCA AT-3′ and 5′- AAA GGA TCC TCA CCT GCG CAG CA- 3′. The product was placed between the NdeI and BamHI cut sites of a modified pET-28 plasmid. The resulting pDR28-*glgE* encodes a recombinant GlgE enzyme possessing an N-terminal histidine tag. The sequence of the *glgE* expression plasmid was confirmed by DNA sequencing.

A gene encoding the *Streptomyces coelicolor* GlgEI-V279S variant protein was placed between the NdeI and XhoI cut sites of a modified pET-32 plasmid. The final pDR32-*glgEI-V279S* plasmid encodes the *S. co glgEI* with a C-terminal histidine tag. The sequence of this construct was verified using DNA sequencing.

### Protein expression and purification

The pDR28-*glgE* plasmid was used to transform T7 Rosetta cells. The bacterial cells were cultured at 37 °C in Luria Broth to an O.D. of 0.6 at 600 nm. Protein expression was induced by the addition of IPTG to a final concentration of 1 mM. Cells were harvested by centrifugation after incubating for 24 hours at 16 °C. Pelleted cells were re-suspended in buffer A containing 20 mM Tris pH 7.5, 5 mM imidazole, 500 mM NaCl, 10% glycerol, and 5 mM β-mercaptoethanol. Lysozyme (10 μM) and DNaseI (100 μM) were added to the cell re-suspension and incubated for one hour on ice prior to lysis by sonication. The resulting suspension was centrifuged at 15,000 *g* for 30 minutes. The supernatant was applied to a 5 mL HiTrap Talon Crude column (GE Healthcare) that had been equilibrated with buffer A. Proteins were eluted from the column via a linear gradient of imidazole from 5–150 mM over 20 column volumes. Fractions containing GlgE were pooled, concentrated by ultrafiltration and applied to a Hi-Load Superdex 200 size exclusion column (GE Healthcare) equilibrated with a buffer containing 20 mM Tris pH 7.5, 150 mM NaCl, 1 mM MgCl_2_, and 0.3 mM TCEP. Fractions containing GlgE were subsequently pooled. The purity of the protein was confirmed using SDS-PAGE.

The pDR32-*glgEI-V279S* plasmid was used to transform BL21* cells. The resulting protein expression and purification procedures are identical to the Mtb GlgE except for one change; the buffer used during size exclusion chromatography contained 20 mM Tris pH 7.5, 150 mM NaCl, and 0.3 mM TCEP.

### Crystallization of the Mtb GlgE-MAL complex

GlgE at 5.3 mg/mL using a 0.1% Abs value of 1.908 was used for crystallization experiments. Maltose was added to the concentrated GlgE to a final concentration of 10 mM. Crystals of GlgE were grown by the hanging-drop vapor diffusion method. Crystallization drops containing 2 μL of GlgE solution and 2 μL of well solution were equilibrated with 100 μL of well solution. Initial crystals were found in a condition containing 0.1 M HEPES pH 7.5 and 1.0 M ammonium formate after roughly 1 month. Improved crystals were produced by the addition of 0.4 μL of either 10% 1,2 butanediol or 40% v/v 2,2,2, trifluoroethanol to the crystallization drop. 1 μL of glycerol was added to the crystallization drop prior to flash-cooling the crystals in liquid nitrogen.

### Soaked Mtb GlgE-M6 complex

Crystals of the Mtb GlgE-MAL complex were produced as previously described. To produce crystals containing maltohexaose, these GlgE crystals were soaked with maltohexaose (10 mM) for 1 hour prior to flash-cooling the crystals in liquid nitrogen in preparation for diffraction experiments. One μL of glycerol was added to the crystallization drop prior to flash-cooling of crystals in liquid nitrogen.

### Crystallization of Sco GlgEI-V279S-MCP and GlgEI-V279S-DDGIM

Sco GlgEI-V279S at 8.0 mg/mL using a 0.1% Abs value of 1.526 was used for crystallization experiments. MCP and the DDGIM were added to the concentrated GlgE to final concentrations of 18 mM and 36 mM respectively. Crystals of Sco GlgEI-V279S were grown by the hanging-drop vapor diffusion method. Crystallization drops containing 2 μL of protein solution and 2 μL of well solution were equilibrated with 100 μL of well solution containing 0.2 M sodium citrate pH 7.0 and 10% PEG 3350. Ethylene glycol was added to a final concentration of 25% to the drop prior to flash-cooling.

### Diffraction experiments

X-ray diffraction experiments were carried out at the LS-CAT beamline at the Advanced Photon Source of Argonne National Labs, Argonne, IL. Mtb GlgE structures were solved using diffraction data collected at a wavelength of 0.97856 Å while the Sco GlgEI-V279S structures were elucidated using diffraction data collected at a wavelength of 1.07819 Å. Diffraction data were integrated and scaled using HKL3000^25^. Initial indexing of the Mtb GlgE diffraction data was not unambiguous. These data were integrated using all possible Bravais lattices and subsequently scaled. The highest symmetry space group that produced R_sym_ values less than 50% were of the C2 space group. Following scaling, the C2 diffraction data were subjected to analysis using the Xtriage tool in PHENIX. This analysis indicated no evidence of twinning^26^.

### Structure Determination for the Mtb GlgE and Sco GlgE-V279S complexes

The structures of the Mtb GlgE-MAL and Sco GlgEI-V279S-MCP were phased using molecular replacement in PHENIX^27^. The Mtb GlgE-MAL and Sco GlgEI-V279S-MCP structures used the monomer of the Sco GlgEI (3ZT5) as a search molecule. The GlgE-M6 structure used the GlgE-MAL structure as a model for rigid body refinement. The Sco GlgEI-V279S-DDGIM structure used the Sco GlgEI-V279S-MCP structure as a model for rigid body refinement. Rigid body refinement, positional and B-factor refinements were carried out using PHENIX^26^,^28^. Following sufficient improvement of the Mtb GlgE model, simulated annealing was performed at 2500 K to minimize any model bias in the refined structure^26^,^28^. Manual refinement of all structures was performed using COOT^29^. Ligand and ligand restraints were generated using eLBOW software in PHENIX^30^. Structural validation was performed using Molprobity^31^.

## Additional Information

**Accession numbers**: The RCSB accession numbers for the GlgE-MAL: 4U33 and GlgE-M6: 4U3C. The RCSB accession numbers for the Sco-V279S-GlgE-MCP: 4U31, and Sco-V279S-GlgE-DDGIM: 4U2Y.

**How to cite this article**: Lindenberger, J. J. *et al.* Crystal structures of Mycobacterium tuberculosis GlgE and complexes with non-covalent inhibitors. *Sci. Rep.* **5**, 12830; doi: 10.1038/srep12830 (2015).

## Figures and Tables

**Table 1:** X-ray crystallographic data collection and refinement statistics.

	Mtb GlgE-MAL	Mtb GlgE-M6	Sco GlgEI-MCP	Sco GlgEI-DDGIM
Data collection				
Wavelength (Å)	0.980	0.980	1.08	1.08
Space group	C2	C2	P4_1_2_1_2	P4_1_2_1_2
Unit Cell dimensions				
*a*	343.2 Å	338.4 Å	113.6 Å	113.8 Å
*b*	242.6 Å	239.4 Å	113.6 Å	113.8 Å
*c*	243.7 Å	239.4 Å	315.1 Å	314.1 Å
α	90°	90°	90°	90°
β	135.1°	134.1°	90°	90°
γ	90°	90°	90°	90°
Resolution (Å)	50.0 – 3.3	50.0 – 3.9	50.0 – 1.9	50.0 – 2.5
*R*_merge_	11.7 (77.6)	18.2 (94.7)	9.0 (61.4)	8.8 (46.6)
*I*/σ*I*	15.0 (2.5)	13.8 (2.2)	37.6 (4.6)	31.3 (3.8)
Completeness (%)	99.9 (100)	99.7 (98.8)	100 (100)	94.0 (94.5)
Redundancy	6.6 (6.3)	7.2 (6.5)	13.4 (13.2)	10.3 (10.4)
Refinement				
Resolution (Å)	47.6 – 3.3	43.7 – 4.0	42.7 – 1.9	47.6 – 2.5
No. unique reflections	210,465	122,284	175,910	69,481
*R*_work/_ *R*_free_	0.1937/0.2220	0.2235/0.2564	0.1643/0.1943	0.1769/0.2165
No. atoms				
Protein	31458	31398	10359	10276
Ligand/ion	138	540	100	44
Water			1447	272
B-factors (Å^2^)				
Protein	72.3	117.4	24.5	51.8
Ligand/ion	83.8	125.2	21.8	45.9
Water			31.8	47.3
R.m.s deviations				
Bond lengths (Å)	0.016	0.008	0.009	0.010
Bond angles (°)	1.41	1.65	1.08	1.37
Ramachandran				
Favored (%)	96.2	95.7	98.0	98.1
Outliers (%)	0.9	1.1	0.1	0.1

The necessary data were obtained from one crystal. Values in parentheses are for the highest shells.
